# Prognostic and immune microenvironment of a cancer-associated fibroblast-related genes signature for biochemical recurrence in prostate cancer

**DOI:** 10.1097/MD.0000000000045451

**Published:** 2025-10-31

**Authors:** Meng Zhang, Min Min, Pan Zhang, Lingxun Li, Weiyang He, Lingxin Wang

**Affiliations:** a Department of Urology, the Third People’s Hospital of Mianyang (Mental Health Center of Sichuan Province), Mianyang, China; b Department of Urology, The First Affiliated Hospital of Chongqing Medical University, Chongqing, China.

**Keywords:** China, Department of Urology, Mianyang, The Third People’s Hospital of Mianyang

## Abstract

Prostate cancer (PCa) ranks among the most prevalent malignancies worldwide. Within the tumor microenvironment (TME), cancer-associated fibroblasts (CAFs) play a crucial role in influencing tumor evolution and progression. To elucidate their prognostic significance, we extracted and integrated PCa data from The Cancer Genome Atlas and the GSE70768, GSE70769, and GSE116918 datasets. Differentially expressed CAF-related genes between normal and tumor tissues were identified, and their associations with CAF subtypes and clinicopathological characteristics were explored through Gene Ontology and Kyoto Encyclopedia of Genes and Genomes enrichment analyses. Based on these features, we constructed a CAF-related prognostic model using multivariate Cox and least absolute shrinkage and selection operator regression analyses. A 9-gene signature (*LMCD1, CXCL2, UNC5B, THBS2, JAM3, PIGR, SCUBE2, SRD5A2*, and *PCGEM1*) was identified to generate a CAFs score for predicting biochemical recurrence risk. Further analyses of the TME, genetic mutations, and drug sensitivity revealed that this signature was closely associated with tumor immunity and treatment response. Collectively, this model highlights the pivotal role of CAFs in shaping the TME and provides novel insights for prognostic prediction and therapeutic strategies in PCa.

## 1. Introduction

Prostate cancer (PCa) poses a global health challenge. Its incidence has been on the rise, driven by factors like aging populations and socioeconomic development.^[1]^ Currently, PCa ranks as one of the most common urological malignancies worldwide, imposing substantial burdens on public health and economies.^[2]^ Various therapeutic strategies for PCa exist, including surgical interventions, endocrine treatments, and radiochemotherapy.^[3,4]^ Despite surgical interventions such as laparoscopy and robotic-assisted radical prostatectomy, biochemical recurrence (BCR) postsurgery remains a clinical challenge. Approximately one-fourth to one-half of patients will experience biochemical recurrence (BCR) after radical therapy, which is defined by the return of measurable prostate-specific antigen levels.^[5,6]^ This high incidence of BCR emphasizes the urgent need for more accurate prognostic models to predict recurrence risk and guide treatment strategies. Hence, it is imperative to identify and establish gene-based prognostic models for the BCR of PCa patients and explore novel therapeutic strategies and targets.

The tumor microenvironment (TME) is a dynamic and complex system that plays a vital role in influencing tumor development and progression.^[7]^ It consists of various cellular and noncellular components, including stromal and immune cells, endothelial cells, cancer cells, the extracellular matrix (ECM), signaling molecules, and bioactive factors.^[8]^ Among the stromal cell types, cancer-associated fibroblasts (CAFs) are the most abundant within the TME.^[9]^ CAFs, which are critical components of the tumor’s ECM, secrete factors that promote cell invasion, angiogenesis, and ECM remodeling.^[10,11]^ An increasing body of evidence, both in vivo and in vitro, suggests that CAFs can accelerate the progression of PCa, sustaining its growth and leading to castration resistance and bone metastasis.^[12,13]^ Emerging studies have also highlighted the role of CAFs in influencing the tumor immune microenvironment, which is critical in tumor progression and therapeutic outcomes. These findings suggest that targeting CAFs could provide a potential therapeutic strategy to overcome drug resistance, BCR following radical surgery, and metastasis.

In this study, we employed various bioinformatics techniques to construct a gene set associated with CAFs for predicting the risk prognosis of BCR in PCa. Additionally, we explored the relationships between a CAFs-related genes (CRGs)-risk score (RS) and the TME, drug resistance, and tumor immune infiltration. Our findings potentially offer a novel and effective predictive marker for personalized treatment in patients with PCa.

## 2. Materials and methods

### 2.1. Data acquisition

For our study, we collected RNA-seq, clinical data, copy number variations, and mutation annotation format (MAF) files for 499 PCa samples and 52 normal samples. These data were extracted from The Cancer Genome Atlas (TCGA; https://portal.gdc.cancer.gov/) database. Additionally, RNA-seq and clinical information from patients with PCa were procured from the Gene Expression Omnibus (GEO; datasets GSE70768, GSE70769, and GSE116918; https://www.ncbi.nlm.nih.gov/geo/) database. By combining gene expression data from the TCGA-PCa cohort and the aforementioned GEO datasets, a comprehensive cohort was assembled. To mitigate batch effects, the “Combat” algorithm was employed. Our refined dataset comprised 1028 patients with PCa for further analysis. In addition to these data sources, immunohistochemical images of human PCa and normal prostate tissues were acquired from the Human Protein Atlas (http://www.proteinatlas.org) database.

### 2.2. Integrated cluster analysis of CAFs-related gene patterns in patient cohorts

To identify genes associated with CAFs, we performed a keyword search using “CAFs” in the publicly accessible Gene Set Enrichment Analysis (GSEA) database. From this search, ~48 CRGs were identified. Additionally, we extracted 49 distinct CAFs-associated genes from a previous study,^[14]^ culminating in a total of 97 CRGs.

### 2.3. Delineation of distinct CRGs clustering profiles

By evaluating the expression of CRGs, combined with the Bayesian information criterion and consensus cluster analysis, we determined the ideal cluster count (*k*) using the Consensus Cluster Plus v3.17 software. Subsequently, patients were divided into 2 subtypes based on their respective *k*-values.

### 2.4. Characterizing and analyzing differentially expressed CRGs (DE-CRGs)

We employed the “limma” package in R to discern candidate DE-CRGs between normal tissues and those from PCa patients, adopting stringent criteria of “|log2(fold-change)| > 1” and an adjusted *P*-value < 0.05. Subsequently, to gain insights into the biological relevance of these DE-CRGs, enrichment analyses were conducted utilizing both the Gene Ontology (GO) terms and the Kyoto Encyclopedia of Genes and Genomes (KEGG) pathways. Furthermore, to ascertain variations in the activities of distinct pathways or biological processes, we implemented the “Gene Set Variation Analysis” (GSVA) package in R.

### 2.5. Development and empirical validation of a prognostic model based on CRGs in oncological settings

We explored the relationship between CRGs and patient prognoses using data from the TCGA-PCa and GEO cohorts. Initially, employing the “survival” R package, we conducted a univariate Cox regression analysis to discern DE-CRGs associated with PCa patient survival, at a significance threshold of *P* < .05. Patients were then stratified into 2 gene clusters (A and B), and evenly split into training (n = 433) and validation (n = 433) cohorts. We then applied the least absolute shrinkage and selection operator (LASSO) regression analysis to select key differentially expressed genes and reduce overfitting. Based on these selected genes, we created a prognostic model consisting of 9 key CRGs. The risk score (CRGs-RS) was calculated using the formula: CRGs-RS = Σ (Exp_i_ * Coef_i_), where Coef_i_ represents the risk coefficient and Exp_i_ denotes gene expression. Based on this risk score, patients with PCa were allocated into either a high-risk group (HRG) or a low-risk group (LRG). The “survminer” R package was used to do a Kaplan–Meier (KM) survival analysis, allowing us to contrast the BCR between the 2 risk groups, with subsequent validation across all data sets.

### 2.6. Assessment of immune cell penetration within CRGs-related regions in patients with PCa

We applied the “ESTIMATE” algorithm to derive immune and stromal scores for patients with PCa. Using the “CIBERSORT” R package, we determined proportional scores for 23 immune cell subtypes in tumor samples, enabling a detailed analysis of immune cell infiltration patterns.

### 2.7. Comprehensive assessment of the interplay between CRGs-RS, TME, genomic alterations, and chemotherapeutic responsiveness

Utilizing the “ESTIMATE” algorithm, we calculated stromal and immune scores for patients with PCa. Proportional scores for 23 immune cell subtypes were determined using the “CIBERSORT” R package, based on RNA levels. We further explored the association between CRGs-RS and tumor mutational burden (TMB), with visualization achieved via the “maftools v2.12.0” R package. To assess patient response to chemotherapy agents, we consulted the genomics of drug sensitivity in cancer database. IC_50_ values were computed with the “pRRophetic” R package.

### 2.8. Ethical considerations

This systematic review obtained all data from publicly accessible databases and did not involve direct contact with individual patients. Consequently, no ethical approval or informed consent was required.

### 2.9. Statistical analysis

All statistical analyses were conducted using R v4.2.2 and Perl v5.30.0.1-64bit software. For group comparisons, we employed the Wilcoxon test, Kruskal–Wallis test, *t*-test, and one-way analysis of variance, based on data distribution. Correlation strengths were determined through Spearman rank correlation and distance correlation. The robustness of our model was ascertained using the receiver operating characteristic (ROC) curve. The optimal survival cutoff associated with the CAFs-score was determined using the “Survminer” package. Survival outcomes were analyzed using KM curves and the Log-rank test to compare group differences. Using the univariate Cox regression, we calculated the hazard ratio for CAFs regulators and associated genes. The CAFs-score, in conjunction with CAFs-related clinical metrics, underwent a multivariate Cox regression to assess its independence as a prognostic indicator. All tests were two-tailed, with significance established at *P* < .05.

## 3. Results

### 3.1. Genetic and transcriptional variations in CRGs within PCa

**Figure 1.**
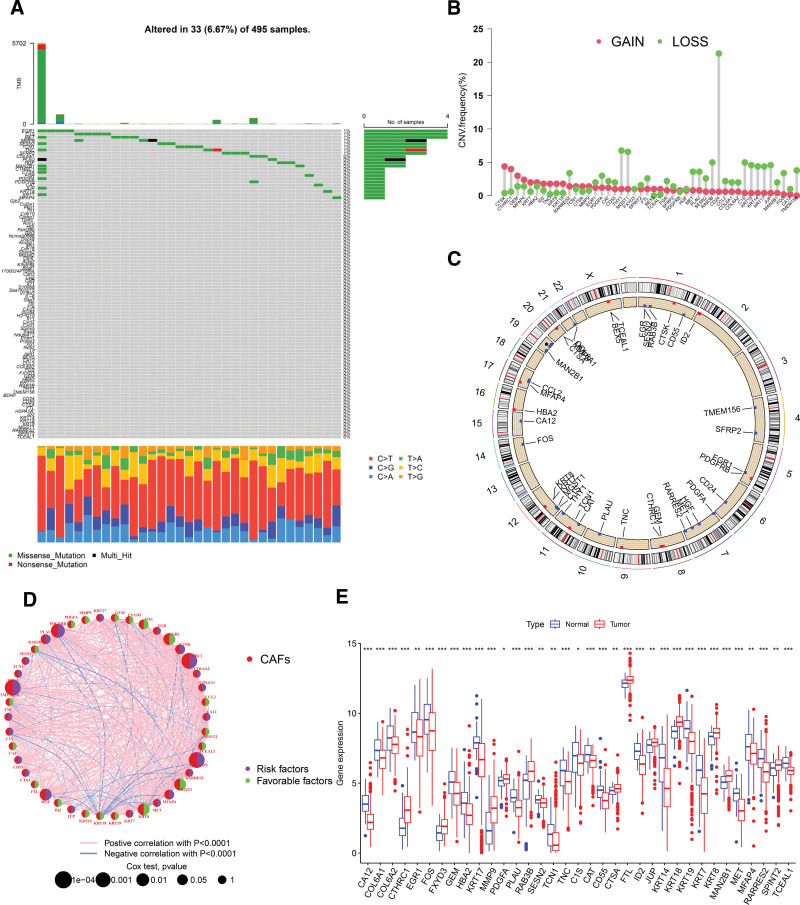
Genetic and transcriptional profiling of CAFs-associated genes in PCa. (A) Somatic mutations of CRGs in the TCGA-PCa dataset. (B) CNV frequency in CRGs among patients with PCa. (C) Chromosomal positioning of CRGs-associated CNVs. (D) Interaction network of CRGs for patients with PCa. (E) Expression disparity of 35 CRGs between normal and PCa tissues.*P < .05, **P < .01, ***P < .001. CAFs = cancer-associated fibroblasts, CNV = copy number variation, CRGs = CAFs-related genes, PCa = prostate cancer, TCGA = the Cancer Genome Atlas.

We investigated the incidence of somatic mutations across 97 CRGs in PCa, revealing mutations in 33 (6.97%) of the 495 PCa samples from the TCGA database (Fig. 1A). Additionally, we analyzed the prevalence of copy number variations and observed a pronounced CNV gain in *CTSK, CTHRC1*, and *GEM* (Fig. 1B). The chromosomal distribution of these CNV alterations is depicted in Figure 1C. A network analysis was performed to provide an integrated view of CRGs interactions and their prognostic implications for PCa (Fig. 1D). Notably, significant associations were identified between numerous CRGs and patient outcomes. A comparative study of CRGs expression in PCa and normal tissues revealed differential expression in 35 of the 97 genes examined (Fig. 1E). Specifically, elevated expressions were observed for genes such as *CTHRC1, MMP9, PDGFA*, and others, while *CA12, COL6A1*, and *EGR1* showed diminished expression. Collectively, our findings underscore marked genetic and expression variations in CRGs between tumor and normal tissues, emphasizing the pivotal role of CRGs in PCa progression.

### 3.2. Delineation and functional profiling of CAFs subtypes in PCa

**Figure 2.**
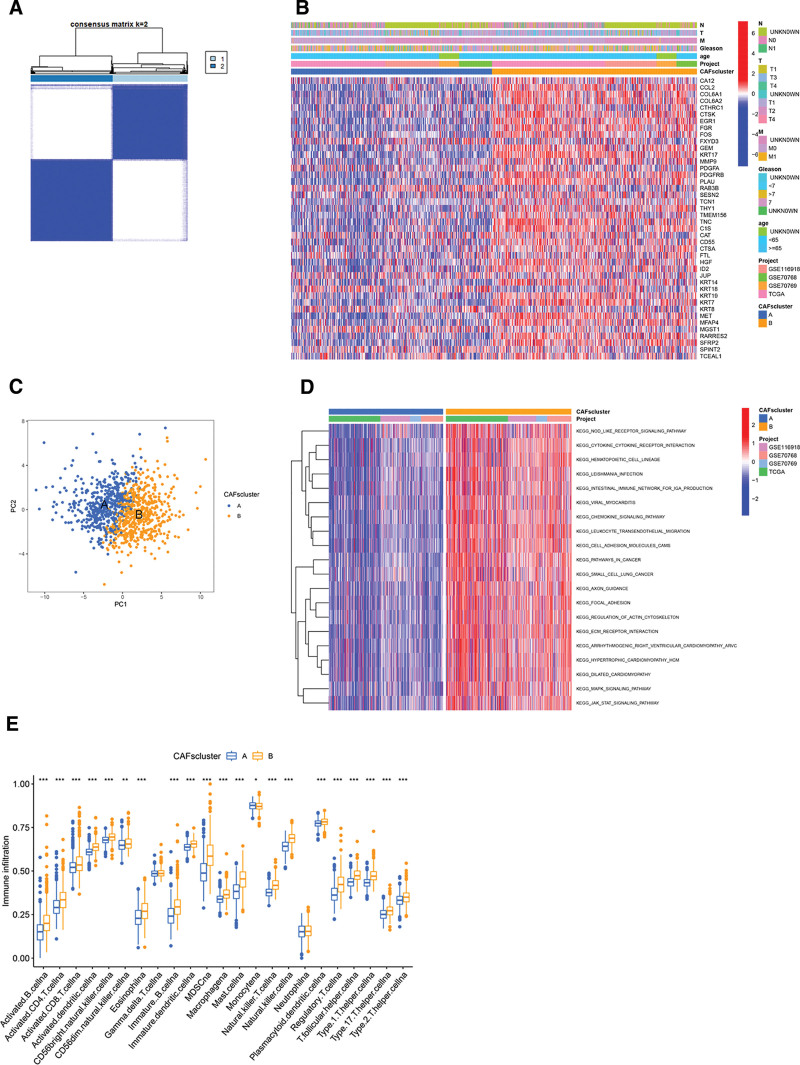
Categorization of CAFs subtypes and their clinical implications in patients with prostate cancer. (A) Consensus clustering distinguishes patients into 2 subtypes. (B) Clinical trait correlation heatmap for the 2 subtypes. (C) Principal component analysis of the clusters. (D) GSVA pathway enrichment across subtypes. (E) Immune cell differential infiltration. CAFs = cancer-associated fibroblasts, GSVA = gene set variation analysis.

We integrated the datasets from TCGA-PCa, GSE70768, GSE70769, and GSE116918 to form a combined cohort of 1028 patients. Consensus clustering on the expression of 97 CRGs was conducted, leading to the classification of patients into 2 subtypes: cluster A (490 patients) and cluster B (538 patients) as visualized in Figure 2A. A heatmap delineated the expression patterns of the 97 CRGs along with associated clinical patient details. Notably, clinical features such as age, Gleason score, and TNM stages were observed in cluster B (Fig. 2B).

A distinct transcriptional profile differentiation between the 2 groups was evident from the principal component analysis in Figure 2C. Subsequently, through GSVA, we discerned differences in biological functionalities between the clusters. Cluster B exhibited significant enrichment in pathways associated with ECM dynamics, including “cytokine-cytokine receptor,” “focal adhesion,” and “ECM receptor interaction” (Fig. 2D). Using the “ssGSEA” algorithm, we quantified tumor-infiltrating immune cells within the clusters. Cluster B displayed notable infiltration of activated lymphocytes, specifically activated B cells, CD4 + T cells, natural killer T cells, and immature B cells. In contrast, cluster A showed a heightened presence of monocytes (Fig. 2E).

### 3.3. Comprehensive analysis of DE-CRGs features in targeted cohorts

**Figure 3.**
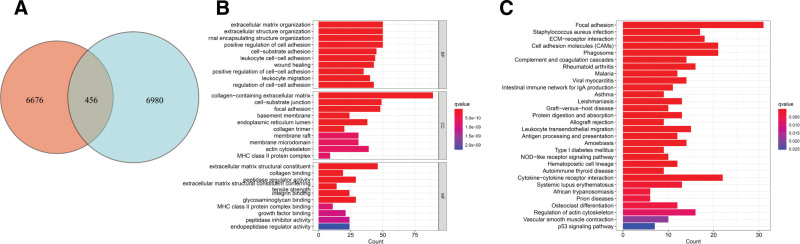
Insights into CAFs-driven differentially expressed genes (DEGs). (A) Venn diagram illustrating DEGs between subtypes. (B) GO enrichment bubble plot for CRGs. (C) KEGG pathway enrichment bar graph. CAFs = cancer-associated fibroblasts, CRGs = CAFs-related genes, GO = gene ontology, KEGG = Kyoto Encyclopedia of Genes and Genomes.

Utilizing the “limma” R package, we systematically probed the functional domains and associated pathways of the 2 CAFs subtypes. Setting stringent parameters, i.e., “|log2(fold-change)| ≥ 1” and “FDR < 0.05,” we identified 456 DE-CRGs that distinguish the subtypes (Fig. 3A). Next, comprehensive GO term and KEGG pathway enrichment analyses were performed on these DE-CRGs. Within the GO biological processes, pronounced enrichment was observed related to ECM orchestration, coordination of external structures, and cell adhesion processes. For GO cellular components, a strong association with the collagen-rich ECM was observed. For GO molecular functions, the significant role of DE-CRGs as -MF dimension earmarked their pivotal contribution as architectural elements of the ECM was revealed (Fig. 3B). Furthermore, KEGG pathway analysis indicated a clear affinity towards focal adhesion pathways (Fig. 3C). These findings suggest that CAFs play a substantial role in shaping the composition of the ECM, which in turn modulates cellular interactions and motility dynamics, as well as enhances tumor metastasis and invasive potential.

### 3.4. Development and verification of a CRGs-based prognostic model

**Figure 4.**
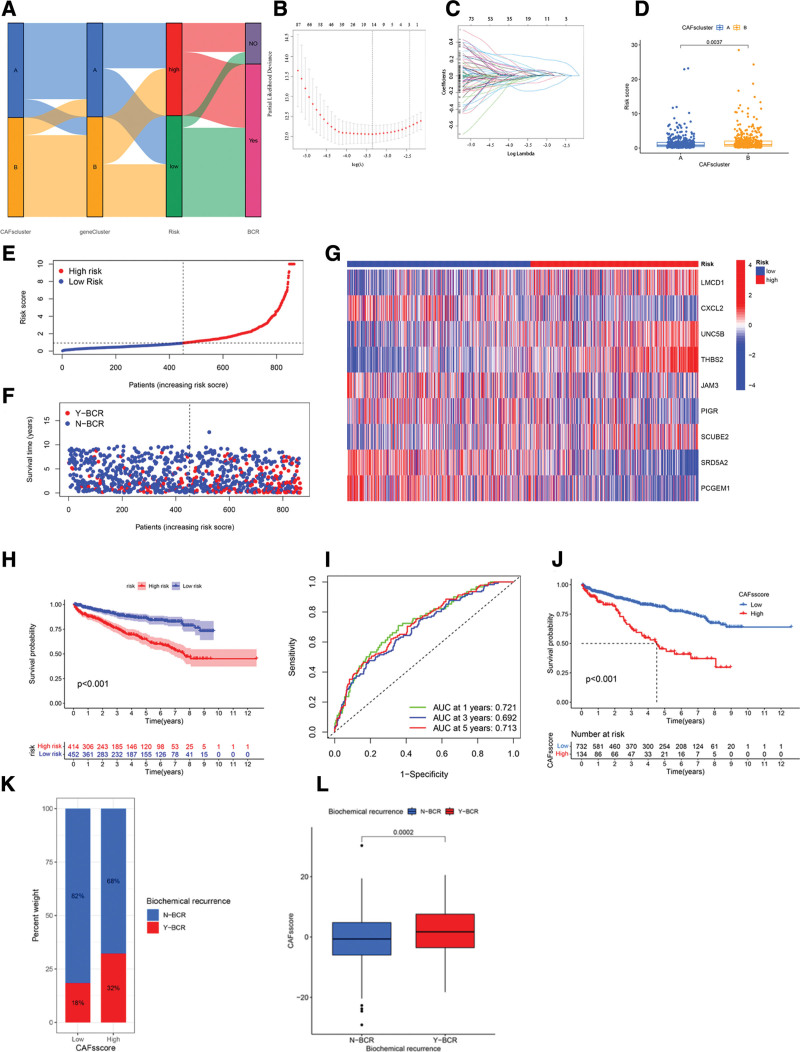
Development and assessment of the CRGs prognostic model. (A) Sankey diagram showing the distribution of CAFs subtypes. (B and C) LASSO regression analysis of CRGs. (D) Cluster changes in risk groups. (E–H) Risk analysis of the entire dataset. (I) BCR rates at 1, 3, and 5 years in the entire dataset. (J) Kaplan–Meier survival analysis of CAF score and BCR risk. (K and L) The relationship between CAFs score and BCR. BCR = biochemical recurrence, CAFs = cancer-associated fibroblasts, CRGs = CAFs-related genes, LASSO = least absolute shrinkage and selection operator.

We derived the CRGs-RS from DE-CRGs. The Sankey diagram illustrated the interplay between the 2 CAFs subtypes, gene clusters, CRGs-RS, and patient BCR outcomes (Fig. 4A). Subsequently, we evenly partitioned the dataset into training (n = 434) and testing (n = 434) cohorts. LASSO regression analysis was applied to the 456 differentially expressed genes to identify 9 CRGs as prognostic signatures (Fig. 4B and C). A subsequent multivariate Cox regression analysis further validated these CRGs. Notably, the identified CRGs comprised 4 high-risk genes (*LMCD1, UNC5B, THBS2*, and *SCUBE2*) and 5 low-risk genes (*CXCL2, JAM3, PIGR, SRD5A2*, and *PCGEM1*). The CRGs-RS was computed as follows: CRGs-RS = (0.4131)*LMCD1 + (–0.2286)*CXCL2 + (0.2771)*UNC5B + (0.4459)*THBS2 + (–0.2848)*JAM3 + (–0.1714)*PIGR + (0.1956)*SCUBE2 + (–0.2908)*SRD5A2 + (–0.0996)*PCGEM1. Notably, distinct CRGs-RS values distinguished the 2 clusters: cluster A exhibited a lower CRGs-RS compared to the elevated score in cluster B (Fig. 4D). Patients were subsequently stratified into HRG and LRGs based on the median CRGs-RS. Our analysis revealed a direct correlation between patient BCR rate and their CRGs-RS (Fig. 4E and F). Specifically, an escalation in CRGs-RS was linked with increased expression of *LMCD1, UNC5B, THBS2*, and *SCUBE2* (Fig. 4G). BCR disparities were evident between the 2 risk groups in the consolidated dataset (*P* < .001; Fig. 4H). Additionally, an ROC curve evaluated the prognostic efficacy of CRGs-RS. The analysis yielded AUC values of 0.721, 0.692, and 0.713 for 1-, 3-, and 5-year BCR rates, respectively (Fig. 4I). Patients in the LRG displayed markedly better BCR outcomes compared to the HRG, underscoring the prognostic robustness of CRGs-RS for PCa (Fig. 4J). The histogram and boxplots indicate that patients with elevated scores exhibit a notably increased rate of BCR compared to those with lower scores (*P* < .001; Fig. 4K and L). This suggests the potential of CRGs-RS as a predictive model for PCa patient outcomes.

### 3.5. Validation of the CRGs-based prognostic model using independent training and testing cohorts

**Figure 5.**
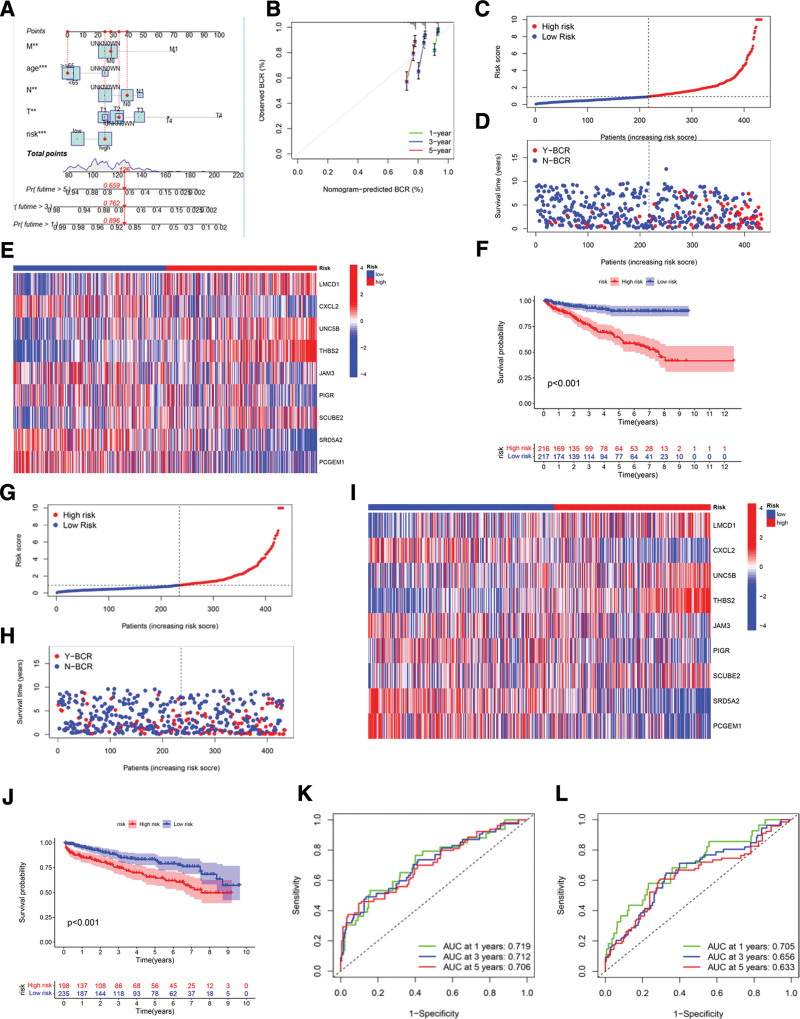
CRGs prognostic model validation in training and testing cohorts. (A–E) Risk analysis, BCR, and predictive accuracy in the training set; (F–J) risk analysis, BCR, and predictive accuracy in the test set; (K and L) ROC curves evaluating the predictive performance of the model at 1, 3, and 5 years in the training and test sets. BCR = biochemical recurrence, CAFs = cancer-associated fibroblasts, CRGs = CAFs-related genes, ROC = receiver operating characteristic.

We constructed a nomogram integrating CRGs-RS and clinical parameters to project the 1-, 3-, and 5-year BCR rates for patients with PCa (Fig. 5A). The calibration curves depicted a robust alignment between the observed and anticipated outcomes (Fig. 5B).

Subsequently, the efficacy of the CRGs prognostic model was evaluated for its predictive precision in both the training (Fig. 5C–F) and validation (Fig. 5G–J) cohorts. For gauging the model’s sensitivity and specificity, ROC curves were employed, focusing on 1-, 3- and 5-year BCR rates for patients with varying RS in both the training cohort (Fig. 5K) and testing set (Fig. 5L). In the training cohort, the AUC values for 1-,3- and 5-year BCR rates were 0.719, 0.712, and 0.706, respectively. In alignment, in the testing set, the AUC values were 0.705, 0.656, and 0.633, respectively. These results demonstrated the consistency and reliability of the CRGs prognostic model in predicting BCR for patients with PCa.

### 3.6. Comprehensive evaluation of the TME in divergent patient cohorts

**Figure 6.**
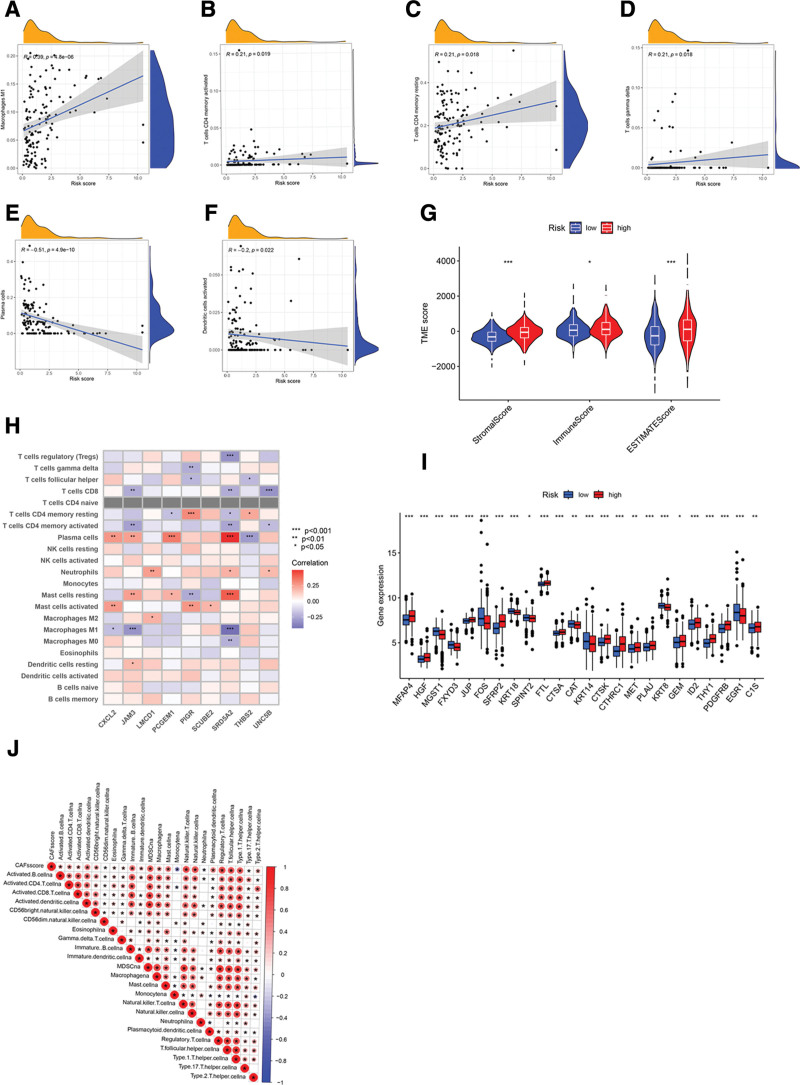
TME landscape in divergent risk categories. (A–F) CRGs-risk score correlation with specific immune cells. (G) CRGs-risk score associated with immune and stromal metrics. (H) Key CRGs links with immune fraction. (I) CRGs expressions across risk groups. (J) Immune cell infiltration correlation.*P < .05, **P < .01, ***P < .001. CAFs = cancer-associated fibroblasts, CRGs = CAFs-related genes, TME = tumor microenvironment.

We harnessed the “CIBERSORT” algorithm to discern correlations between the CRGs-RS and the relative abundance of immune cells. We found a direct association between high CRGs-RS and the presence of M1 macrophages, CD4 + memory-activated T cells, CD4 + memory-resting T cells, and gamma delta T cells, indicating a higher abundance of these immune cells in patients with high CRGs-RS. Conversely, activated dendritic cells and plasma cells showed a negative association with high CRGs-RS (Fig. 6A–F), suggesting a lower presence of these immune cells in patients with high CRGs-RS.

To elucidate the TME status in patients with PCa, we computed stromal, ESTIMATE, and immune scores. Interestingly, patients in the HRG exhibited markedly elevated stromal and ESTIMATE scores compared to those in the LRG, indicating a higher presence of stromal and immune cells within the primary TME (Fig. 6G). Additionally, we assessed the correlations between our prognostic model, based on 9 CRGs, and various immune cell proportions. The results revealed a significant interplay between these 9 CRGs and diverse immune cell populations (Fig. 6H). Further exploration of the expression profiles in the 2 risk groups showed that the LRG group had elevated expression of *MGST1, FOS, KRT18, CAT, KRT14, KRT8*, and *EGR1*, while the HRG group exhibited increased expression of other CRGs (Fig. 6I).

Lastly, by applying single-sample GSEA (ssGSEA) to the infiltrating immune cells in the TME, we identified significant pathway alterations (Fig. 6J). Notably, there was a significant inverse correlation between CAFs-RS and both monocytes and neutrophils. This suggests a potential role of CAFs in modulating the immune microenvironment in PCa.

### 3.7. The association of CRGs-RS with genetic mutations and pharmacological responsiveness

**Figure 7.**
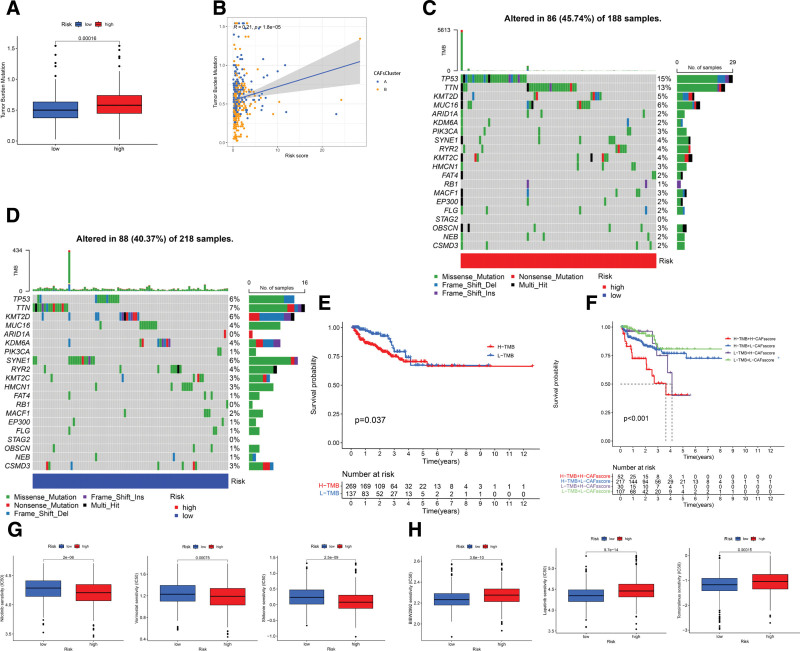
Exploring CRGs-risk correlations: mutation, tumor mutation burden (TMB), and chemotherapeutic response. (A–B) The Boxplot and Spearman correlation shows that patients in HRG had a higher TME rate. (C–D) Mutation landscape in both risk groups. (E–F) Survival analysis using Kaplan–Meier curves for patients with prostate cancer based on TMB and CAF score. (F–G) Chemotherapeutic sensitivity profiles. CAF = cancer-associated fibroblast, CRGs = CAFs-related genes, HRG = high-risk group, TME = tumor microenvironment.

In the TCGA-PCa dataset, we analyzed the TMB profiles. Strikingly, the HRG exhibited a significantly higher TMB relative to the LRG (*P* < .001; Fig. 7A). Spearman analysis revealed a positive correlation between CRGs-RS and TMB (*R* = 0.21, *P* < .001; Fig. 7B). We then explored the somatic mutation landscape in both risk groups. Predominant mutations were seen in genes such as *TP53, TTN, KMT2D, MUC16*, and *SYNE1* (Fig. 7C and D). KM plots demonstrated a significant BCR disparity between high and low TMB groups, with the high TMB group displaying reduced survival (Fig. 7E; *P* < .05). An integrated analysis of BCR, factoring in both TMB and CAFs-scores, indicated the worst prognosis for patients with high TMB and CAFs-scores (Fig. 7F; *P* < .001). Lastly, we delved into the relationship between CRGs-RS and chemotherapeutic drug responsiveness. Enhanced drug sensitivity, specifically to vinblastine, camptothecin, and parthenolide, was evident in the LRG. In contrast, the HRG demonstrated increased IC_50_ values for agents like temsirolimus, BIBW2992, and lapatinib (Fig. 7G).

### 3.8. Immunohistochemical visualization of DE-CRGs in normal prostate and PCa tissues

**Figure 8.**
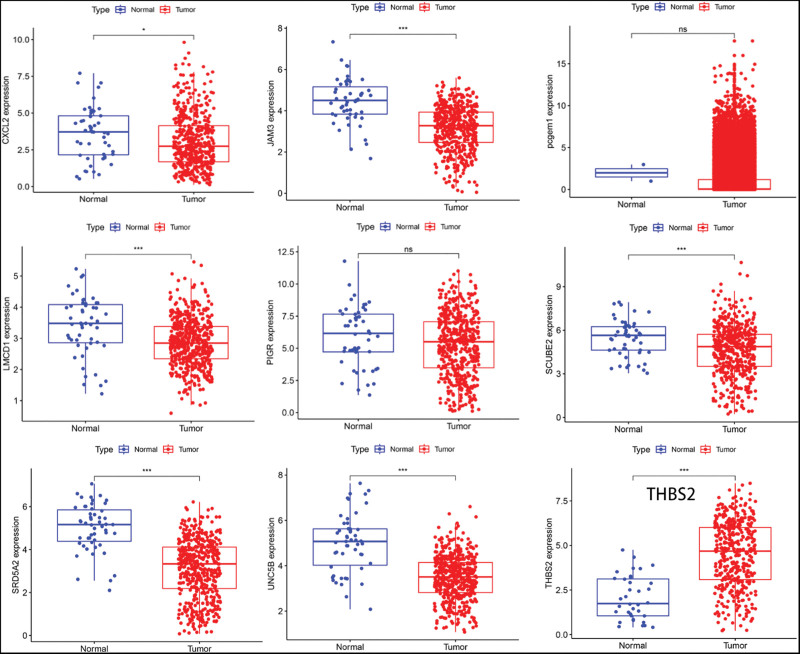
The expression of genes in the prognostic model was compared between prostate cancer tissues and normal tissues in the TCGA database.*P < .05, **P < .01, ***P < .001. TCGA = the Cancer Genome Atlas.

Using the TCGA dataset, we performed a single-gene analysis on CRGs. It was discerned that *LMCD1, JAM3, CXCL2, UNC5B, SCUBE2*, and *SRD5A2* had markedly higher expression in normal prostate tissues than in the PCa specimens (*P* < .05). Conversely, *THBS2* was more prominently expressed in PCa samples relative to normal tissues (*P* < .001). Notably, *PIGR* and *PCGEM1* displayed no significant expression differences between the 2 groups (*P* > .05; Fig. 8).

**Figure 9.**
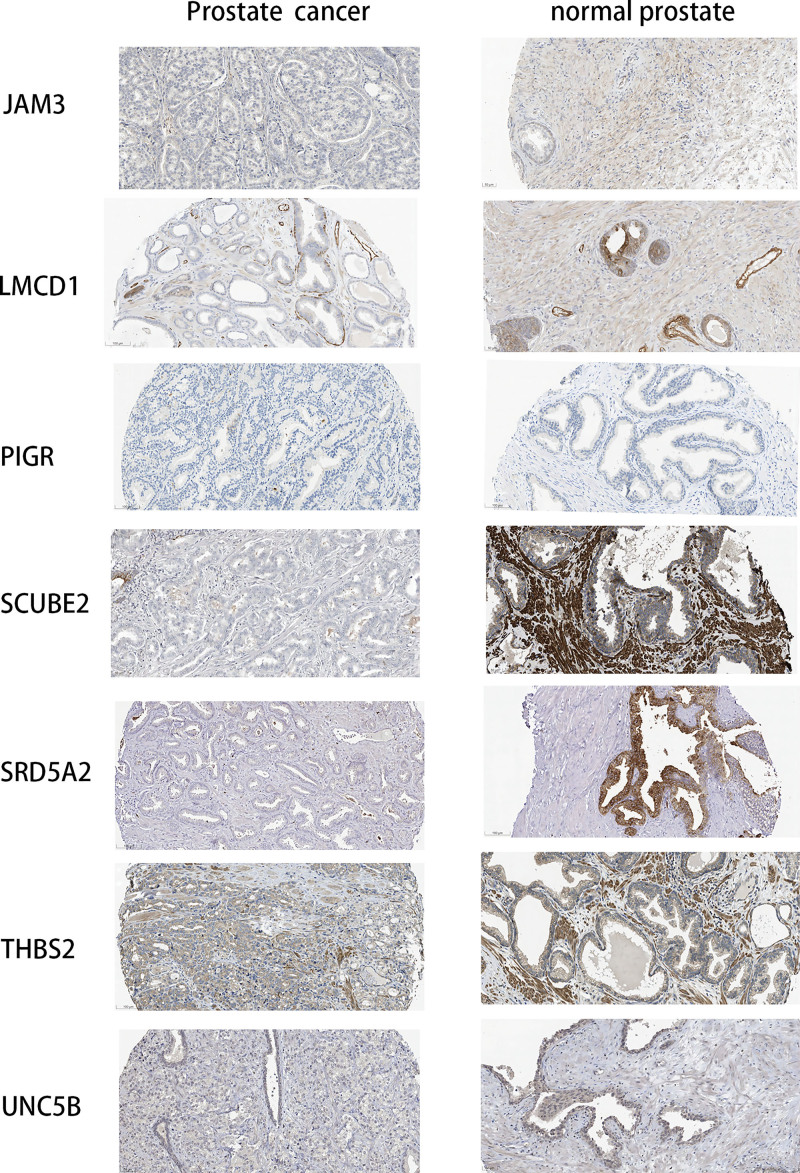
IHC-derived CRGs expression in healthy and prostate cancer tissues. CAFs = cancer-associated fibroblasts, CRGs = CAFs-related genes, IHC = immunohistochemistry.

In addition to *CXCL2* and *PCGEM*, the differential expression between normal prostate and PCa tissues for the remaining CRGs was further validated using the Human Protein Atlas database (Fig. 9). These findings corroborate the differential expression patterns of these genes observed in the TCGA dataset, reinforcing their relevance in PCa.

## 4. Discussion

Our evolving understanding of PCa, as one of the most common malignancies in men, has underscored the pivotal role played by the TME beyond the mere presence of malignant cells.^[15]^ The TME, comprising cellular, acellular, and signaling components, profoundly influences the initiation, progression, metastatic potential, and therapeutic responsiveness of prostate tumors.^[16]^ Within the TME, CAFs are particularly abundant and an influential component in various malignancies, including PCa. CAFs can arise from a variety of cellular origins, including resident fibroblasts, mesenchymal stem cells, and even endothelial and epithelial cells through processes like mesenchymal transition. They exert their effects on PCa cells through both paracrine signaling and direct cell–cell interactions.^[17]^ CAFs secrete key factors, such as TGF-β, FGF, and HGF, which have been identified to promote prostate tumor cell proliferation, migration, and invasion.^[18–20]^ Concurrently, these factors can shape the surrounding TME by facilitating angiogenesis and modulating the immune landscape in a manner that often favors tumor evasion from immune surveillance. Emerging evidence suggests that targeting CAFs may provide a promising strategy to enhance tumor immunotherapy and overcome drug resistance. Targeting CAFs and their interactions could offer new avenues for therapeutic interventions in PCa.

In the present investigation, we delineated the impact of CRGs on the risk of BCR in PCa. We conducted a comprehensive analysis using genetic and clinical datasets from the TCGA and GEO databases to focus on genes associated with CAFs. We meticulously developed a prognostic model to predict BCR following prostatectomy, utilizing a combination of univariate COX regression and LASSO regression techniques. Through a consensus clustering approach, we identified discrete patient subgroups and isolated 9 pivotal CRGs for our prognostic model: *LMCD1, CXCL2, UNC5B, THBS2, JAM3, PIGR, SCUBE2, SRD5A2*, and *PCGEM*. These genes emerged as crucial indicators, shedding light on the molecular intricacies of the disease’s progression. A CRGs-RS was subsequently developed, revealing insights into principal component analysis characteristics, and demonstrating robust performance in predicting BCR, drug sensitivity, TMB, and immune cell infiltration in patients with PCa. This CAFs-RS also revealed significant correlations with patient prognosis and highlights the dynamic alterations in CAFs during tumorigenesis and PCa progression, underscoring the integral role of CAFs in tumor evolution within the TME.

Consensus clustering algorithms are instrumental in effectively discerning patient clusters with varied characteristics within expansive datasets. Utilizing this unsupervised approach, we discerned 2 unique molecular subtypes predicated on the expression profiles of 9 CAFs-related regulators. These subtypes manifested marked immune activation with concomitant activation of pertinent immune pathways. Through GSVA enrichment analysis, we probed the differential biological behaviors between the subtypes. Specifically, subtype B was notably enriched in pathways linked to the ECM and tumor invasion, with a pronounced emphasis on ECM–receptor interaction and focal adhesion. Existing literature corroborates the role of the ECM receptor in PCa progression and its association with unfavorable outcomes.^[21,22]^ Proteins related to focal adhesion have been independently implicated as indicators of adverse clinical prognosis in PCa.^[23]^ In a study by Pan et al, single-cell RNA sequencing across 34 PCa samples delineated 2 distinct fibroblast subgroups, namely myofibroblast-like CAFs and inflammatory CAFs.^[24]^ Our observations resonate with this categorization, as we also effectively divided patients into 2 clusters based on the expression of CRGs.

For our prognostic risk model, we identified 9 pertinent CRGs markers. Notably, *CXCL2, JAM3, PIGR, SRD5A2*, and *PCGEM1* demonstrated a favorable prognostic association, whereas *LMCD1, UNC5B, THBS2*, and *SCUBE2* exhibited an inverse relationship with prognosis. In the complex landscape of PCa genomics, several genes stand out for their potential implications in tumorigenesis and progression. CXCL2, a chemokine, orchestrates the TME and when overexpressed in prostate malignancies, augments tumor cell migration – highlighting its potential role in metastasis.^[25]^ JAM3, integral to cellular junctions, mediates adhesion and communication, and while the broader JAM family has indications in oncogenesis, the specific role of JAM3 in prostate malignancies requires further elucidation.^[26]^ In the context of tumorigenesis, the *PIGR* gene has been implicated in various cancers, where its expression may be associated with tumor progression, metastasis, and patient prognosis.^[27]^ Alterations in *PIGR* expression can impact the TME, potentially influencing cancer cell growth, immune evasion, and treatment responses.^[28]^ The enzyme SRD5A2 is central to testosterone metabolism, and together with its genetic variants may influence PCa susceptibility, highlighting its diagnostic and therapeutic potential.^[29]^ PCGEM1, characterized by its prostate-centric expression, correlates heightened activity with reduced patient survival, suggesting prognostic relevance.^[30]^ LMCD1, identified across various cancers, emerges as a potential biomarker.^[31]^ Chang et al reported that somatic mutations in *LMCD1* facilitated cell migration and metastasis in hepatocellular carcinoma.^[32]^ UNC5B’s involvement in axonal guidance hints at its engagement in prostate tumor development and spread.^[33]^ The long noncoding RNA UNC5B-AS1 has been reported to promote the malignant progression of PCa through competitive binding with caspase-9.^[34]^ THBS2, a multifunctional glycoprotein within the ECM, plays a pivotal role in cellular interactions, adhesion, and migration.^[35]^ Chen et al showed that thrombospondin-2 enhances bone metastasis in PCa by elevating matrix metalloproteinase-2 levels via miR-376c downregulation.^[36]^ Lastly, the *SCUBE2* gene, an integral member of the SCUBE family, has garnered considerable attention in recent oncological research. Not only has it been identified as a potential tumor suppressor in certain malignancies, but its aberrant expression patterns have also been correlated with tumor progression and patient prognosis in various cancer types.^[37]^ Wu et al, through single-cell sequencing, identified that *SCUBE2* facilitates bone metastasis in luminal breast cancer by altering immune-suppressive osteoblastic niches.^[38]^ Collectively, these genes offer profound insights into the molecular intricacies of PCa, and a deeper exploration can drive innovative therapeutic strategies.

Through an extensive examination, we observed that categorization based on a CAFs-score could provide distinctions in the clinicopathological characteristics, immune landscape, TME, BCR, TMB, and drug sensitivity in patients with PCa. Although the current model exhibits strong performance in retrospective datasets, it is important to recognize that our findings were derived entirely from in silico analyses. The absence of direct experimental validation (e.g., through in vitro or in vivo studies) limits the immediate translational relevance of the model. Future biological investigations are warranted to validate the mechanistic roles of the identified genes in CAF-mediated PCa progression and to assess the potential for clinical application.

## 5. Conclusion

In our investigation, we formulated and substantiated a risk model based on 9 CRGs, furnishing preliminary insights into the prediction of BCR in PCa. Moreover, this model offers the potential to forecast drug responsiveness and delineate the immunological profile of patients with PCa. The identified genes hold promise in steering PCa immunotherapeutic strategies and anticipating BCR occurrences.

## Acknowledgments

We extend our gratitude for the substantial data contributions from the TCGA and GEO projects. Acknowledgments is also made to Bullet Edits Limited for their invaluable linguistic editing and proofreading of the manuscript.

## Author contributions

**Data curation:** Ling Xin Wang.

**Formal analysis:** Min Min, Pan Zhang.

**Investigation:** Ling Xun Li.

**Validation:** Wei Yang He.

**Writing – original draft:** Meng Zhang.
